# RPLP1, an NS4B-interacting protein, enhances production of CSFV through promoting translation of viral genome

**DOI:** 10.1080/21505594.2022.2033500

**Published:** 2022-02-07

**Authors:** Longxiang Zhang, Jihui Lin, Maoyang Weng, Ying Wen, Yanming Zhang, Wen Deng

**Affiliations:** a College of Veterinary Medicine, Northwest A&F University, Yangling, Shaanxi, China; b School of Nursing, Southwest Medical University, Luzhou, Sichuan, China

**Keywords:** RPLP1, classical swine fever virus, NS4B, interaction, translation

## Abstract

Classical swine fever virus (CSFV), the etiological agent of classical swine fever (CSF), causes serious financial losses to the pig industry. Using yeast two-hybrid screening, we have previously identified ribosomal protein RPLP1 as a potential binding partner of CSFV NS4B. In this study, the interaction between host RPLP1 and CSFV NS4B was further characterized by co-immunoprecipitation (co-IP), glutathione S-transferase (GST) pulldown, and confocal microscopy. In addition, lentivirus-mediated shRNA knockdown of RPLP1 drastically attenuated CSFV growth, while stable overexpression of RPLP1 markedly enhanced CSFV production. Moreover, cellular RPLP1 expression was found to be significantly up-regulated along with CSFV infection. Dual-luciferase reporter assay showed that depletion of RPLP1 had no effects on the activity of CSFV internal ribosome entry site (IRES). In the first life cycle of CSFV, further studies revealed that RPLP1 depletion did not influence the intracellular viral RNA abundance but diminished the intracellular and extracellular progeny virus titers as well as the viral E2 protein expression, which indicates that RPLP1 is crucial for CSFV genome translation. In summary, this study demonstrated that RPLP1 interacts with CSFV NS4B and enhances virus production via promoting translation of viral genome.

## Introduction

Classical swine fever (CSF), a highly contagious disease of pig caused by classical swine fever virus (CSFV), is also one of the notifiable infectious diseases to the World Organization for Animal Health (OIE) and has inflicted serious financial burden to the pork industry around the world since outbreak [1]. CSFV is a non-segmented, single-stranded, positive-sense and enveloped RNA virus, belonging to the genus *Pestivirus* within the family *Flaviviridae* [2]. The genome of CSFV is 12.3 kb in length and is composed of 5′- untranslated regions (UTR) and 3′- UTR in flanks and a big open reading frame (ORF) encoding a polyprotein in center. The central polyprotein is further cleaved into 4 structural proteins (Core, E^rns^, E1 and E2) and 8 nonstructural (NS) proteins, including N^pro^, p7, NS2, NS3, NS4A, NS4B, NS5A, and NS5B by proteases derived from the host or virus [3,4]. The structure of UTRs in CSFV genome is different from that of most eukaryotic mRNAs. The 5′-UTR lacks the terminal cap but possesses an important structure, namely internal ribosome entry site (IRES), whose essential function is to mediate the translation initiation of viral genomes [5]. The 3′-UTR misses a poly(A) tail, whereas includes a variable AU-enriched motif and a conservative region, which is primarily involved in regulating the process of replication [6].

CSFV NS4B is a membrane-associated multifunction viral protein, which has been shown to restrain the activation of the toll-like receptor 3 (TLR3) signaling pathway, and acts positively in viral anti-apoptosis process [7,8]. Mutation in the C-terminal TIR-like domain of NS4B leads to virulence attenuation and reduction of virus growth [9]. The NTPase activity of NS4B is critical for virus replication and infectious virion production [10]. Furthermore, NS4B-interacting host proteins are involved in different physiological metabolic processes and signaling pathways, such as cargos endocytosis, ribosome biogenesis, ubiquitination-mediated degradation, RIG-I-like receptor signaling pathway, and cytoplasmic DNA sensing signaling pathway [11].

There are approximately 80 ribosomal proteins (RPs) encoded by human genome. In addition to being constituents of the ribosome, RPs play significant parts in biosynthesis of ribosomes via stabilizing rRNAs to fold correctly in assembly of ribosomal subunits [12,13]. Increasing evidences have indicated RPs possess functions beyond ribosomes, which participate in various processes in cells such as growth, development, apoptosis, tumorigenesis, and genomic integrity [13–15]. Moreover, RPs are involved in the lifecycle of diverse viruses, for instance, subjecting phosphorylation to respond viral infection, interacting with viral proteins to facilitate replication, and hijacked by viral IRES to orchestrate translation [16–19]. RPLP1 is one of the elements of the lateral stalk in the 60S ribosomal subunit [20,21] and usually helps to orchestrate the elongation phase of translation [22,23]. Previous studies suggested that RPLP1 is a pivotal host factor that facilitates viral proteins synthesis of Zika virus (ZIKV), dengue viruses (DENV), and yellow fever virus (YFV) [24,25]. Interestingly, RPLP1 was screened as one of the CSFV NS4B binding partners in our previous yeast two-hybrid (Y2H) system [11], however, whether RPLP1 is involved in CSFV infection has not been revealed.

In this work, the interactivity of host RPLP1 with CSFV NS4B was confirmed by co-IP, GST-pulldown, and confocal microscopy. Then, CSFV infection was found to up-regulate expression of RPLP1 for enhancing viral infection. Investigation of the underlying mechanism indicated that the enhancement of RPLP1 on CSFV growth was not caused by modulation of the CSFV IRES efficiency but functioned in translation of CSFV genome.

## Materials and methods

### Cell lines and virus

Porcine kidney cells (PK15) and human embryonic kidney (HEK-293 T) cells were kept in our laboratory [26]. Both cell lines were cultured in DMEM (Gibco, Carlsbad, UK) supplemented with 10% FBS (Gibco) at 37°C in 5% CO_2_. CSFV (Shimen strain; GenBank ID: AF092448) was obtained from the Control Institute of Veterinary Bio-products and Pharmaceuticals (Beijing, China).

### Antibodies

Rabbit anti-RPLP1 polyclonal antibodies (pAbs; #21636-1-AP), mouse anti-β-actin monoclonal antibody (mAb; #66009-1-Ig), mouse anti-GFP mAb (#66002-1-Ig), goat anti-mouse IgG-HRP (#SA00001-1), and goat anti-rabbit IgG-HRP (#SA00001-2) antibody were all obtained from Proteintech (Wuhan, China). Mouse anti-E2 mAb was produced in Abmart (Shanghai, China). Rabbit anti-Flag pAbs (#CW0287M) and rabbit anti-Myc pAbs (#CW00899M) were purchased from CWBIOTECH (Beijing, China). Mouse anti-GST mAb (#sc-138) was obtained from Santa Cruz Biotechnology (Dallas, USA). Rabbit anti-eEF1A1 pAbs (#ab153710) was obtained from Abcam (Cambridge, United Kingdom). Goat anti-mouse IgG-Alexa Fluor 594 pAbs (#A32742) was produced from Thermo Fisher Scientific (Waltham, USA). Anti-pig IgG-FITC pAbs (#F1638) was obtained from Sigma Aldrich (St. Louis, USA).

### Reagents

Phenyl methane sulfonyl fluoride (PMSF; #ST505), proteinase K (#ST533), tritonX-100 (#P0096), WB and IP lysis buffer (#P0013), RIPA lysis buffer (#P0013D), DAPI staining solution (#C1005), 4% paraformaldehyde (PFA; #P0099), BeyoECL plus (#P0018S), enhanced BCA protein assay kit (#P0010S), and enhanced cell counting kit-8 (CCK-8; #C0041) were produced from Beyotime Biotechnology (Shanghai, China). TurboFect transfection reagent (#R0533), GST protein interaction pull-down kit (#21516), and OPP protein synthesis assay kits (#C10457) were obtained from Thermo Fisher Scientific. RNAiso plus reagent (#9108Q), viral RNA/DNA extraction kit (#9766), Prime Script RT reagent kit (#RR047B), and TB Green fast qPCR mix (#RR430A) were obtained from TAKARA (Dalian, China). Dual-luciferase reporter assay system (#E1910) was obtained from Promega (Madison, USA). Puromycin (#P8833), cholesterol (#C4951), cycloheximide (#239763-M), hexadimethrine bromide (#H9268), and L-α-Phosphatidylcholine (#P3556) were obtained from Sigma Aldrich. Chemically defined lipid concentrate (#11905-031) and advanced DMEM (#12491-015) were obtained from Invitrogen (Carlsbad, USA).

### Plasmid construction

The cDNA of CSFV NS4B was inserted into the pcDNA3.1(+) and pEGFP-C1 to construct pcDNA3.1-NS4B-Flag and pEGFP-NS4B, respectively. The cDNA of CSFV NS5A was also inserted into the pcDNA3.1(+) to create pcDNA3.1-NS5A-Flag. 5′-UTR of CSFV was amplified and cloned into pGL4.21[*Luc2P*/Puro] to generate pGL4.21-IRES according to previous research [27]. The coding sequence of RPLP1 (GenBank ID: DQ629169.1) from PK15 cells was inserted into pCDH-CMV-MCS-EF1-copGFP-T2A-Puro, pDsRed-N1, pcDNA3.1(+), and pGEX-6P-1 to create pCMV-RPLP1, pDsRed-RPLP1, pcDNA3.1-RPLP1-Myc, and pGST-RPLP1. The cDNA of full-length RPLP1 (aa 1–114), N-terminus (aa 1–66), and C-terminus (aa 67–114) were sub-cloned into the pEGFP-C1 to create pGFP-RPLP1 (1–114), pGFP-RPLP1 (1–66), and pGFP-RPLP1 (67–114), respectively. The coding sequence of eEF1A (GenBank ID: NM_001097418.2) was inserted into pCDH-CMV-MCS-EF1-copGFP-T2A-Puro to create CMV-eEF1A.

The shRNAs targeting RPLP1 and ShN (negative control) were designed on the site (http://rnaidesigner.thermofisher.com/). The annealed double-stranded DNA fragments were inserted into pCDH-U6-MCS-EF1-Green-Puro to create RPLP1-Sh1, RPLP1-Sh2, RPLP1-Sh3, and RPLP1-ShN lentiviral vectors. Primer sequences involved in the current study are shown in Table 1.

**Table 1.** Primers used in this study

Primers	Sequence (5′-3′)	Purpose
NS4B-Flag-F	CCCAAGCTTGCCACCATGGCTCAGGGGGATGTGCAGAGA	Construction of pcDNA3.1-NS4B-Flag
NS4B-Flag-R	CGCGGATCCTTA**CTTATCGTCGTCATCCTTGTAATC**TAGCTGGCGGATCTTTCCTTCA	
RPLP1-Myc-F	CCCAAGCTTGCCACCATGGCTTCCGTCTCGGAG	Construction of pcDNA3.1-RPLP1-Myc
RPLP1-Myc-R	CGCGGATCCTTA**CAGATCCTCTTCAGAGATGAGTTTCTGCTC**GTCAAAAAAACCAAAGCCCAT	
NS5A-Flag-F	CCCAAGCTTGCCACCATGTCAAGTAATTACATTCTAGAGCTCCT	Construction of pcDNA3.1-NS5A-Flag
NS5A-Flag-R	CGCGGATCCTTA**CTTATCGTCGTCATCCTTGTAATC**CAGTTTCATAGAATACACTTTTGC	
GST-RPLP1-F	CGCGGATCCATGGCTTCCGTCTCGGAGC	Construction of pGEX-GST-RPLP1
GST-RPLP1-R	CCGCTCGAGTTAGTCAAAAAAACCAAAGCCCAT	
GFP-NS4B-F	GGAATTCTATGGCTCAGGGGGATGTGC	Construction of pEGFP-NS4B
GFP-NS4B-R	CGGGATCCTTATAGCTGGCGGATCTTTCC	
Red-RPLP1-F	CCCAAGCTTATGGCTTCCGTCTCGGAG	Construction of pDsRed-RPLP1
Red-RPLP1-R	CGCGGATCCCGGTCAAAAAAACCAAAGCCCAT	
RPLP1(1-114)-F	CCGAATTCT**GGTGGCGGGGGCTCT**ATGGCTTCCGTCTCGGAGC	Construction of pGFP-RPLP1(1–114)
RPLP1(1-114)-R	CGCGGATCCTTAGTCAAAAAGACCAAAGCCCATAT	
RPLP1(1-66)-F	CCGAATTCT**GGTGGCGGGGGCTCT**ATGGCTTCCGTCTCGGAG	Construction of pGFP-RPLP1(1–66)
RPLP1(1-66)-R	CGCGGATCCTTAACCAGCCCCCACATTGC	
RPLP1(67-114)-F	CCGAATTCT**GGTGGCGGGGGCTCT**ATGGGACCTGCCCCAGCAGC	Construction of pGFP-RPLP1(67–114)
RPLP1(67-114)-R	CGCGGATCCTTAGTCAAAAAGACCAAAGCCCATAT	
CMV-RPLP1-F	CCGGAATTCTATGGCTTCCGTCTCGGAG	Construction of pCDH-CMV-RPLP1
CMV-RPLP1-R	CGCGGATCCGTCAAAAAAACCAAAGCCCAT	
CMV-eEF1A-F	TGCTCTAGA ATGGGAAAGGAGAAGACTCACA	Construction of pCDH-CMV-eEF1A
CMV-eEF1A-R	ATAAGAATGCGGCCGCTTTAGCCTTCTGAGCTTTCTGAG	
CSFV-IRES-F	CGGGGTACCGTATACGAGGTTAGTTCATTCTC	Construction of pGL4.21-IRES
CSFV-IRES-R	CCCAAGCTTGTGCCATGTACAGCAGAGA	
RPLP1-Sh1-F	GATCCGCTCGCCTGCATTTACTCTGCCAAGAGGCAGAGTAAATGCAGGCGAGCTTTTTG	Knockdown of RPLP1
RPLP1-Sh1-R	AATTCAAAAAGCTCGCCTGCATTTACTCTGCCTCTTGGCAGAGTAAATGCAGGCGAGCG	
RPLP1-Sh2-F	GATCCGGAGGATAAGATCAATGCTCTCAAGAGAGAGCATTGATCTTATCCTCCTTTTTG	Knockdown of RPLP1
RPLP1-Sh2-R	AATTCAAAAAGGAGGATAAGATCAATGCTCTCTCTTGAGAGCATTGATCTTATCCTCCG	
RPLP1-Sh3-F	GATCCGGTGTAAATGTTGAGCCATTCCAAGAGGAATGGCTCAACATTTACACCTTTTTG	Knockdown of RPLP1
RPLP1-Sh3-R	AATTCAAAAAGGTGTAAATGTTGAGCCATTCCTCTTGGAATGGCTCAACATTTACACCG	
ShN-F	GATCCGCTTAAACGCATAGTAGGACTCAAGAGAGTCCTACTATGCGTTTAAGCTTTTTG	Negative control of knockdown
ShN-R	AATTCAAAAAGCTTAAACGCATAGTAGGACTCTCTTGAGTCCTACTATGCGTTTAAGCG	
qRPLP1-F	AATGTCAACATCGGGAGCCT	RT-qPCR for detection of RPLP1 mRNA
qRPLP1-R	TTTGCTTCTACTTTCTTCTCCTCAG	
qCSFV-F	GATCCTCATACTGCCCACTTAC	RT-qPCR for detection of CSFV genome RNA
qCSFV-R	GTATACCCCTTCACCAGCTTG	
qβ-actin-F	CAAGGACCTCTACGCCAACAC	RT-qPCR for detection of β-actin mRNA
qβ-actin-R	TGGAGGCGCGATGATCTT	

Underlines show restriction enzyme sites or loop ring. Bolds show flexible linker or Flag/Myc tag.

### Western blotting (WB)

PK15 cells were lysed using RIPA buffer (containing 1 mM PMSF) on ice for 0.5 h. After centrifugation, the concentration of all the samples were detected using BCA protein assay kit. Samples were separated by 12% SDS-polyacrylamide gel electrophoresis (SDS-PAGE). Then, the gels were electrotransferred onto the polyvinylidene difluoride (PVDF) membranes (Merck Millipore; Darmstadt, Germany). After blocking with 5% skimmed milk, the membranes were incubated with the corresponding antibodies at 4°C overnight. After incubated with goat anti-rabbit or mouse IgG-HRP antibodies at room temperature (RT) for 2 h, the membranes were subsequently washed with TBST (tris-buffered saline containing 0.05% Tween 20). Finally, the target proteins were detected using ECL reagent and imaged using Tanon-410 gel imaging system (Tianneng; Shanghai, China). The relative levels of target proteins were measured and calculated using ImageJ software, and the ratio was exhibited as fold change below the images.

### Co-IP assays

For immunoprecipitation of endogenous RPLP1, PK15 cells transfected with the plasmids pcDNA3.1-NS4B-Flag were lysed by WB and IP lysis buffer (containing 1 mM PMSF) on ice for 0.5 h. Lysates were collected into microcentrifuge tube and centrifugated at 4°C for 0.5 h. The anti-Flag-M2 magnetic beads (#M8823, Sigma Aldrich) were placed in magnetic separator to discard the storage buffer. After washed with TBS, the equilibrated magnetic beads were co-incubated with the supernatant of protein extracts at RT for 2 h with gentle mixing to capture the Flag fusion proteins. Finally, the magnetic beads were collected with magnetic separator and washed five times with TBS followed by detecting target protein with WB.

For exogenous co-IP, HEK-293 T cells co-transfected with the plasmids pcDNA3.1-NS4B-Flag and pcDNA3.1-RPLP1-Myc. Simultaneously, cells co-transfected with pcDNA3.1-NS4B-Flag and pcDNA3.1-Myc as well as pcDNA3.1-RPLP1-Myc and pcDNA3.1-Flag were set as control. At 48 h post transfection (hpt), the cells were lysed and the supernatant was co-incubated with equilibrated Anti-Flag-M2 magnetic beads or anti-c-Myc magnetic beads (#88842, Thermo Fisher Scientific), and the subsequent steps were identical to those of endogenous co-IP assay.

### GST-pulldown assays

HEK-293 T cells transfected with eukaryotic plasmid pcDNA3.1-NS4B-Flag to express CSFV NS4B protein and transfected with empty vector as negative control. The *E. coli* BL21 (DE3) were transformed with prokaryotic plasmid pGEX-6p-1 or pGEX-GST-RPLP1 and induced to express GST or GST-RPLP1 proteins. The GST-RPLP1 or GST proteins were purified by GST-agarose resins (#21516, Thermo Fisher Scientific). The resins were washed and then co-incubated with HEK-293 T cell lysates containing NS4B-Flag at 4°C for 12 h. After washing by elution buffer, the eluted proteins were subjected to WB with rabbit anti-Flag pAbs or rabbit anti-GST mAb.

### Confocal microscopy

PK15 cells grown in confocal dishes were co-transfected with pDsRed-RPLP1 and pEGFP-NS4B (under the condition of CSFV inoculation or not) as well as the empty plasmids. At 48 hpt, the transfected cells were fixed with 4% PFA at RT for 10 min, followed by washing with cold-PBS. The cell nuclei were labeled with DAPI solution away from light. Cell images were acquired using a LSM510 META confocal microscope (Zeiss; Oberkochen, Germany). The co-localization between RPLP1 and NS4B was analyzed by Image-pro plus software and expressed as Pearson’s correlation coefficient.

### Lentiviruses production and stable cell lines construction

The lentiviruses with RPLP1 knockdown or overexpression were produced as previously described [28]. Briefly, HEK-293 T cells within logarithmic growth phase were co-transfected with indicated constructs (CMV-RPLP1 or RPLP1 shRNA) and three assistant vectors (pVSV-G, pRev and pGag/Pol). At 16 hpt, the culture medium was discarded and the fresh advanced DMEM (containing 2% FBS [v/v], cholesterol [0.01 mM], L-α-phosphatidylcholine [0.01 mM], L-glutamine [4.0 mM], and chemically defined lipid [1:1000]) was added. After another 48 h, the recombinant lentiviruses in culture supernatant were collected and concentrated, and HEK-293 T cells were used to estimate the lentiviruses titers by detecting 50% tissue culture infective dose (TCID_50_).

To develop stable cell lines, PK15 cells were incubated with the RPLP1 knockdown or overexpression recombinant lentiviruses at a multiplicity of infection (MOI) of 1 and polybrene was added to facilitate infection. At 12 h post infection (hpi), survival cells were selected with culture medium containing puromycin (6 μg/mL) for a week to generate the stable cell lines.

### Cell viability assay

The viability of cell lines was assessed using the CCK-8. Briefly, the cell lines (RPLP1 knockdown or overexpression) were seeded into 96-well plates with 2000 cells/well to form a full layer. Subsequently, the cell viability solution (10 μL) was added to each well, and the plates were transferred to 37°C incubation for 1–4 h. Absorbance peaks of all the samples were detected at 450 nm using an automated microplate reader (Molecular Devices; San Francisco, USA).

### RNA extraction and quantitative reverse transcription PCR (RT-qPCR)

Total cellular RNA was isolated using the RNAiso plus reagent (#9108Q, TAKARA). The RNA was reversely transcribed into cDNA using the Prime Script RT reagent kit with gDNA Eraser (#RR047B, TAKARA). RT-qPCR was performed with the TB Green Fast qPCR mix (#RR430A, TAKARA) on a 7500 RT-PCR system (Life Technologies; Carlsbad, USA) to detect the levels of RPLP1 mRNA or viral genome RNA. The relative fold changes of indicated genes were evaluated by the 2^−ΔΔCT^ method and normalized to β-actin gene [29]. All the target-specific RT-qPCR primers were designed by the “Primer-BLAST” tool to span an exons-exons junction and listed in Table 1.

### Virus titer determination

Indirect immunofluorescence assay (IFA) was used to determine the CSFV titers according to our previous report [3]. Briefly, PK15 cells seeded in 96-well plates were infected with 10-fold serial dilution of progeny viruses at 37°C for 1 h. After washed away the unbound virus particles, the cells were further incubated for 48 h. Then, the cell monolayers were fixed with 4% PFA and permeated with 0.1% tritonX-100. After washed with PBS, the permeated cells were blocked with 5% skimmed milk and incubated with anti-CSFV pAbs at 4°C overnight. Afterward, the FITC-labeled anti-pig secondary antibody (1:200) was applied to stain at RT for 1 h. Fluorescence images were collected by fluorescence microscopy (Nikon; Tokyo, Japan), and CSFV titers are calculated and expressed as TCID_50_/mL according to the method of Reed-Muench [30].

### Dual-luciferase reporter assay

The established stable cells with RPLP1 knockdown (RPLP1-Sh3), RPLP1 overexpression (CMV-RPLP1), and eEF1A overexpression (CMV-eEF1A) as well as the negative control cells (ShN and CMV) were co-transfected with recombinant pGL4.21-IRES and pGL4.74 [*hRluc/TK*] for 36 h. The cells were lysed and the CSFV IRES luciferase activities were analyzed using a dual-luciferase reporter assay system (#E1910, Promega). Furthermore, the reporter plasmids pGL4.21-IRES and pGL4.74 [*hRluc*/TK] along with pcDNA3.1-NS4B-Flag or with pcDNA3.1-NS5A-Flag were co-transfected into PK15 cells for 36 h, following the same detection method as description above. The CSFV IRES activity was represented by the ratio Firefly/Renilla.

### Virus binding and entry assays

Binding and entry assays of CSFV were carried out as previous reports [31,32]. Briefly, for binding assay, the cell lines with RPLP1 knockdown or overexpression were challenged with CSFV (1 MOI) and then incubated at 4°C for 1 h to allow virions absorb on the surface of cells but not entry. After the unbound virus particles were washed away with PBS containing proteinase K, the RNA abundance of virions bound on cells was measured by RT-qPCR.

For viral entry assay, cell lines mentioned above were incubated with CSFV (1 MOI) at 4°C for 1 h. Subsequently, the inoculated cells were washed and shifted to 37°C for an additional 1 h to allow the bound virus to entry. Then, the medium containing unabsorbed virions were removed and the washed cells were prepared for RT-qPCR analysis.

### *Analysis of total* de novo *protein synthesis*

Total *de novo* protein synthesis assay were implemented using the OPP protein synthesis assay kit (#C10457, Thermo Fisher Scientific). Briefly, the stable cell lines (RPLP1-Sh3 and ShN) grown in six-well plates were labeled with O-propargyl-puromycin (OPP) at 37°C for 0.5 h. Meanwhile, the untreated cells and the cells incubated with CHX (100 μg/mL) at 37°C for 0.5 h before OPP treatment served as negative controls. After incubation, cells were fixed using 4% PFA followed by a permeabilization step using 0.1% tritonX-100 and then washed with PBS. The permeabilized cells were stained with Alexa Fluor-594 picolyl azide at RT for 20 min. Finally, the fluorescence signal was assessed using a flow cytometer (Partec; Münster, Germany) and illustrated in mean fluorescence intensity (MFI).

### Statistical analysis

All data are shown as means ± standard deviations (SD) of three independent experiments. The Student’s t-test was conducted to analyze and calculate the differences between each group with GraphPad Prism 8 (GraphPad Software Inc., La Jolla, USA). A P value < 0.05 was considered to be statistically significant.

## Results

### CSFV NS4B binds to RPLP1

**Figure 1.**
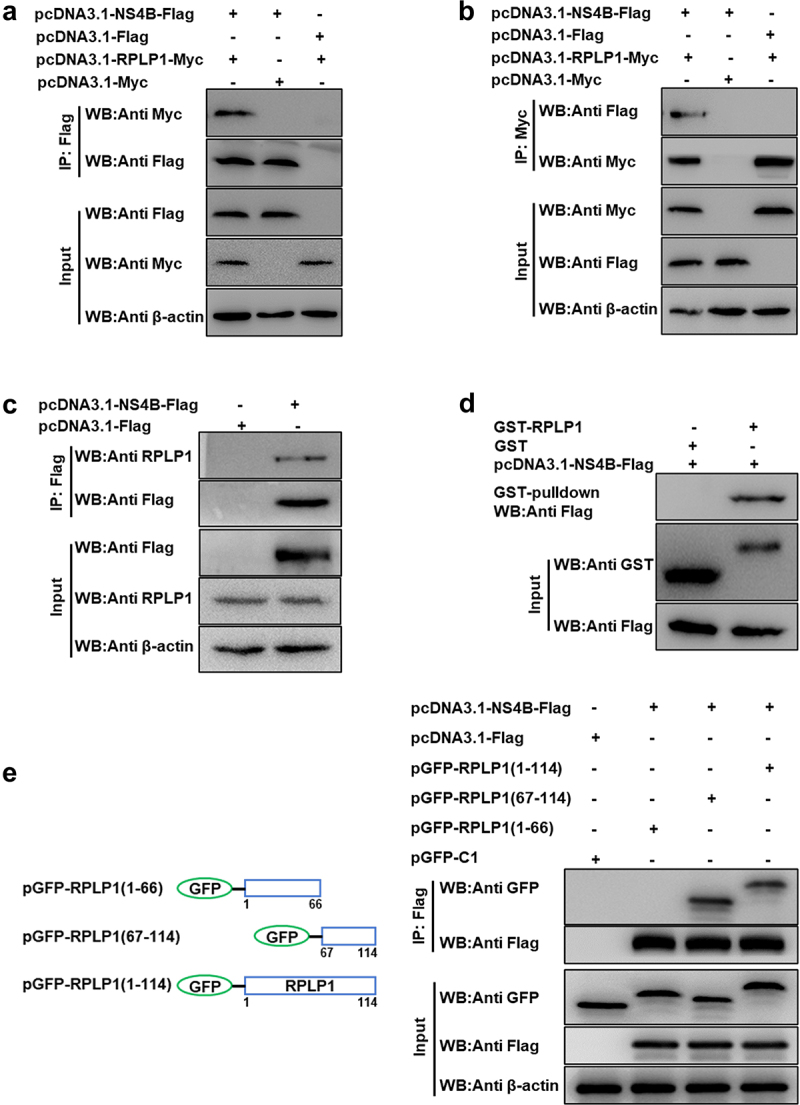
CSFV NS4B interacts with RPLP1.

Our previous Y2H screening identified RPLP1 as a potential binding protein of CSFV NS4B [11]. To substantiate the interaction between NS4B and RPLP1, co-IP assays were carried out with HEK-293 T cells co-expressing RPLP1-Myc and NS4B-Flag, with RPLP1-Myc or NS4B-Flag expressed alone as control. The anti-Flag-M2 magnetic beads were used to immunoprecipitate NS4B-Flag together with its interacting partners from the cell lysates, and WB was applied to detect the proteins binding to NS4B-Flag. Results showed that RPLP1-Myc was precipitated by NS4B-Flag, but no signal was detected from control groups (Figure 1(a)). The reciprocal co-IP assays indicated NS4B-Flag was also precipitated by RPLP1-Myc, and no signal was detected form control (Figure 1(b)). Next, co-IP assay was performed to check whether endogenous RPLP1 could be co-precipitated by NS4B-Flag in PK15 cells. Figure 1(c) displays that endogenous RPLP1 was detected in immunoprecipitates with rabbit anti-RPLP1 pAbs, indicating NS4B binds to cellular RPLP1. This is identical with the above exogenous detection results (Figure 1(a,b)).

(a-b) CSFV NS4B interacted with exogenous RPLP1. HEK-293 T cells were co-transfected with the indicated plasmid combinations or empty vectors for 48 h. NS4B-Flag or RPLP1-Myc were immunoprecipitated from cell lysates and their co-precipitated proteins were subjected to WB and detected with rabbit anti-Flag pAbs or rabbit anti-Myc pAbs. (c) CSFV NS4B bound to endogenous RPLP1. NS4B-Flag was transiently expressed in PK15 cells then immunoprecipitated with anti-Flag pAbs, co-precipitated endogenous RPLP1 was detected with rabbit anti-RPLP1 pAbs. (d) CSFV NS4B bound to the recombinant RPLP1. The GST-RPLP1 recombinant proteins were expressed in prokaryotic cells and purified with GST beads, then incubated with lysate of HEK-293 T cells expressing NS4B-Flag. After washed with cold-PBS, the eluted complexes were subjected to WB and detected with specific antibodies. (e) CSFV NS4B interacted with C-terminal RPLP1. HEK-293 T cells were co-transfected with the pcDNA3.1-NS4B-Flag and pEGFP-RPLP1 (1–114), pEGFP-RPLP1 (1–66) or pEGFP-RPLP1 (67–114) as indicated for 48 h. NS4B-Flag was immunoprecipitated from cell lysates and its co-precipitated GFP fusion proteins were detected with anti-GFP mAb. Left panel showed the schematic diagram of RPLP1 and its truncated constructs.

To further corroborate their interaction, the GST-pulldown assays were carried out. NS4B-Flag protein was expressed in eukaryotic cells, while GST-RPLP1 and GST (as control) proteins were expressed in prokaryotic bacteria and purified using GST-agarose resins from lysates. The purified target proteins were applied to pull down NS4B-Flag. GST-RPLP1 was found to bind to NS4B-Flag, while GST protein was not (Figure 1(d)).

Next, to investigate the functional region of RPLP1 required for interacting with CSFV NS4B, we constructed a GFP-tagged full-length RPLP1 (amino acids [aa] 1 to 114) and two truncations (Figure 1(e), left; N-terminus [aa 1 to 66], C-terminus [aa 67 to 114]) according to the UniProt entry (A1XQU7) as well as the cryo-EM structure information of its human homolog (PDB ID: 4V6X). HEK-293 T cells co-expressing NS4B-Flag together with either GFP-RPLP1 (1–114) or its two truncations were harvested and lysed for co-IP assays. As shown in Figure 1(e), NS4B-Flag specifically bound to the full-length and RPLP1 (67–114). On the contrary, NS4B-Flag could not interact with the RPLP1 (1–66) part, indicating that the aa 67 to 114 within RPLP1 is crucial for its interaction with NS4B-Flag. Collectively, all the results demonstrated that CSFV NS4B indeed binds to host RPLP1.

### CSFV NS4B co-localizes with RPLP1

**Figure 2.**
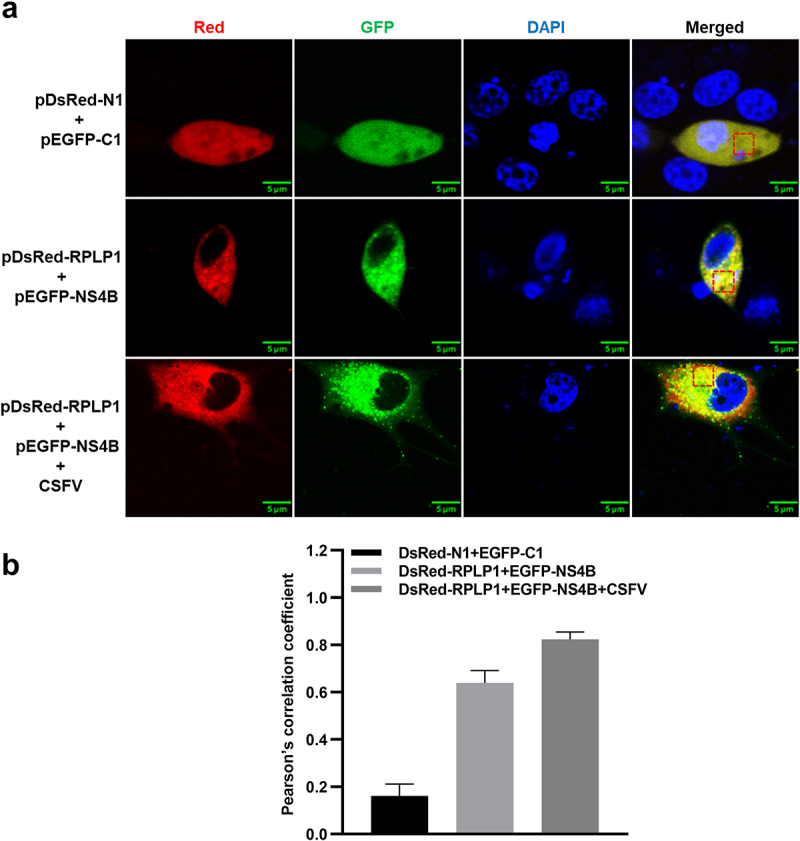
CSFV NS4B co-localizes with RPLP1.

To determine whether NS4B co-localizes with RPLP1, the confocal microscopy was carried out to analyze the distribution of EGFP-NS4B and RPLP1-DsRed in PK15 cells. In Figure 2(a), RPLP1 co-localized with NS4B in cytoplasm (top and middle row). We further checked the protein co-localization in the context of CSFV infection, which was not disturbed by untagged viral NS4B during infection (bottom row). Notably, more EGFP-NS4B granular fluorescent signals were observed in the infected cells than uninfected, resulted by enhanced formation of viral replication/assembly structures on the intracellular membrane. Image analyses with Pearson’s correlation coefficients showed the co-localization of NS4B and RPLP1 was also higher when cells were infected with CSFV (Figure 2(b)), indicating RPLP1 also works together with NS4B in the context of CSFV infection. Together, these results further confirmed the interaction and functional association between CSFV NS4B and host RPLP1.

(a) Representative confocal images of DsRed-RPLP1 and EGFP-NS4B in PK15 cells. PK15 cells were co-transfected with pEGFP-NS4B and pDsRed-RPLP1 in the absence (middle row) or presence (+ CSFV, bottom row) of CSFV infection. Cell nuclei were stained with DAPI, and pEGFP-C1 co-transfected with pDsRed-N1 served as negative control (top row). Scale bar equals to 5 μm. (b) Fluorescence correlation analysis results presented as Pearson’s correlation coefficient. The values were measured by Image-pro Plus software, and shown as mean ± SD of three individual pictures.

### Knockdown of RPLP1 impairs CSFV proliferation

**Figure 3.**
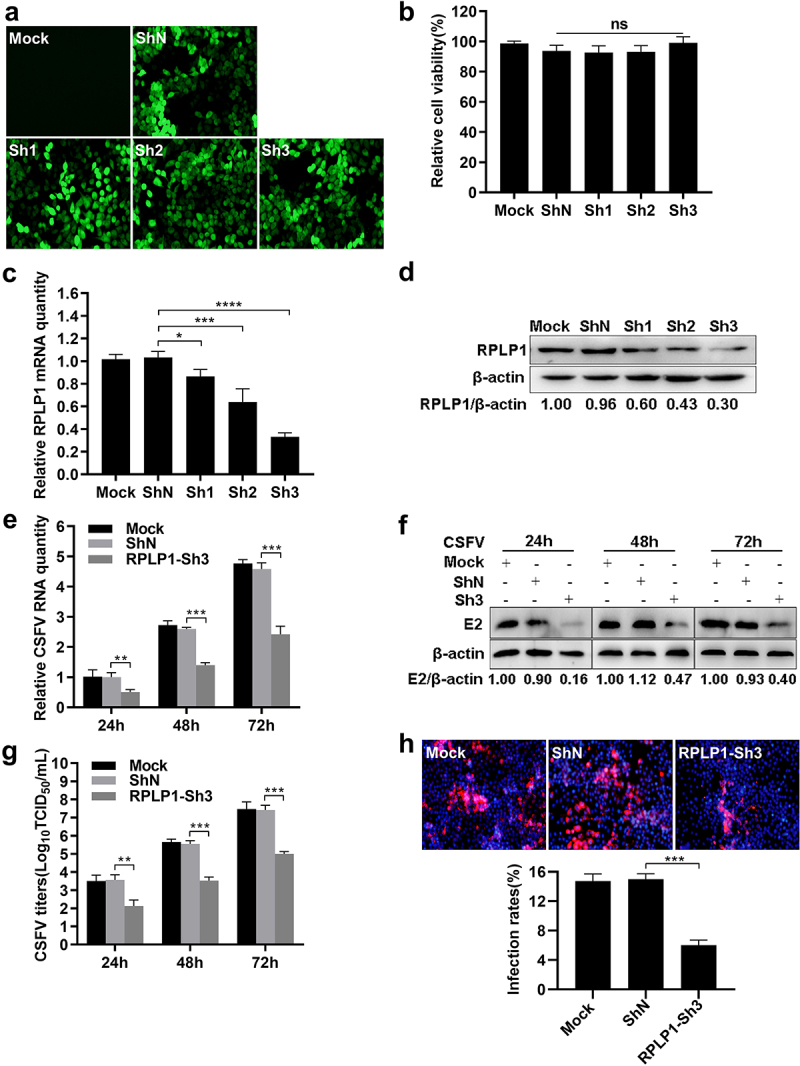
Knockdown of RPLP1 impairs CSFV infection.

To examine the biological significance of RPLP1 during CSFV growth, three pairs of specific shRNAs (Sh1, Sh2, and Sh3) targeting host RPLP1 and a negative control (ShN) were transduced into PK15 cells via recombinant lentiviruses. Expression of each shRNA did not affect cell viability, and Sh3 showed the best knockdown effect on RPLP1 at mRNA levels as well as protein levels (Figure 3(a–d)). Subsequently, RPLP1-Sh3 or ShN cell lines and PK15 cells were infected with CSFV (0.1 MOI). Results showed, compared to the control cells, knockdown of RPLP1 led to observable decrease of viral RNA and E2 protein amount at 24, 48, and 72 hpi (Figure 3(e,f)). Besides, the titers of progeny virus and viral infection rates were tested by IFA, which showed remarkably decreased virus titers and viral infectivity in RPLP1 knockdown cells (Figure 3(g,h)). These results revealed that knockdown of RPLP1 attenuates CSFV proliferation.

(a) Confirmation the shRNAs against RPLP1 (Sh1, Sh2 and Sh3) and ShN expressing cell lines by detection of EGFP reporter. (b) Cell viability of RPLP1 knockdown or ShN cell lines was estimated by CCK-8. (c) The relative RPLP1 mRNA levels in RPLP1 knockdown cell lines were quantified by RT-qPCR. (d) The endogenous RPLP1 expression levels of RPLP1 knockdown cell lines were determined by WB and densitometry. The optical density ratio of RPLP1 to β-actin was calculated and the value of untransfected group (Mock) was normalized to 1.00. (e-g) Assessment of CSFV infection in RPLP1 knockdown or ShN cell lines. Cells were infected with CSFV (0.1 MOI) and collected at 24, 48, and 72 hpi for detection the viral RNA abundance, E2 protein expression, and progeny virus titers. (e) The relative CSFV RNA levels in RPLP1-Sh3 or ShN cell lines were quantified by RT-qPCR. (f) The CSFV E2 protein expression levels in RPLP1-Sh3 or ShN cell lines were determined by WB and densitometry. The optical density ratio of E2 protein to β-actin at each time points were calculated and the values of untransfected group (Mock) were normalized to 1.00. (g) The progeny CSFV titers in RPLP1-Sh3 or ShN cell lines were measured by IFA and expressed as TCID_50_/mL. (h) RPLP1-Sh3 or ShN cell lines were infected with CSFV (0.1 MOI) and the representative immunofluorescence images were captured at 24 hpi. Cell nuclei (blue) were labeled with DAPI, and the CSFV E2 proteins (red) were stained with mouse anti-E2 mAb. The infection rates for CSFV were shown below the images and calculated by quantifying the number of viral E2 expressing cells in total DAPI-staining cells. At least 500 cells for each sample in three independent experiments were counted using ImageJ software. All results are shown as the mean ± SD of three independent experiments. Student’s t-test: * (P < 0.05); ** (P < 0.01); *** (P < 0.001); **** (P < 0.0001); ns, not significant (P > 0.05).

### Overexpression of RPLP1 enhances CSFV proliferation

**Figure 4.**
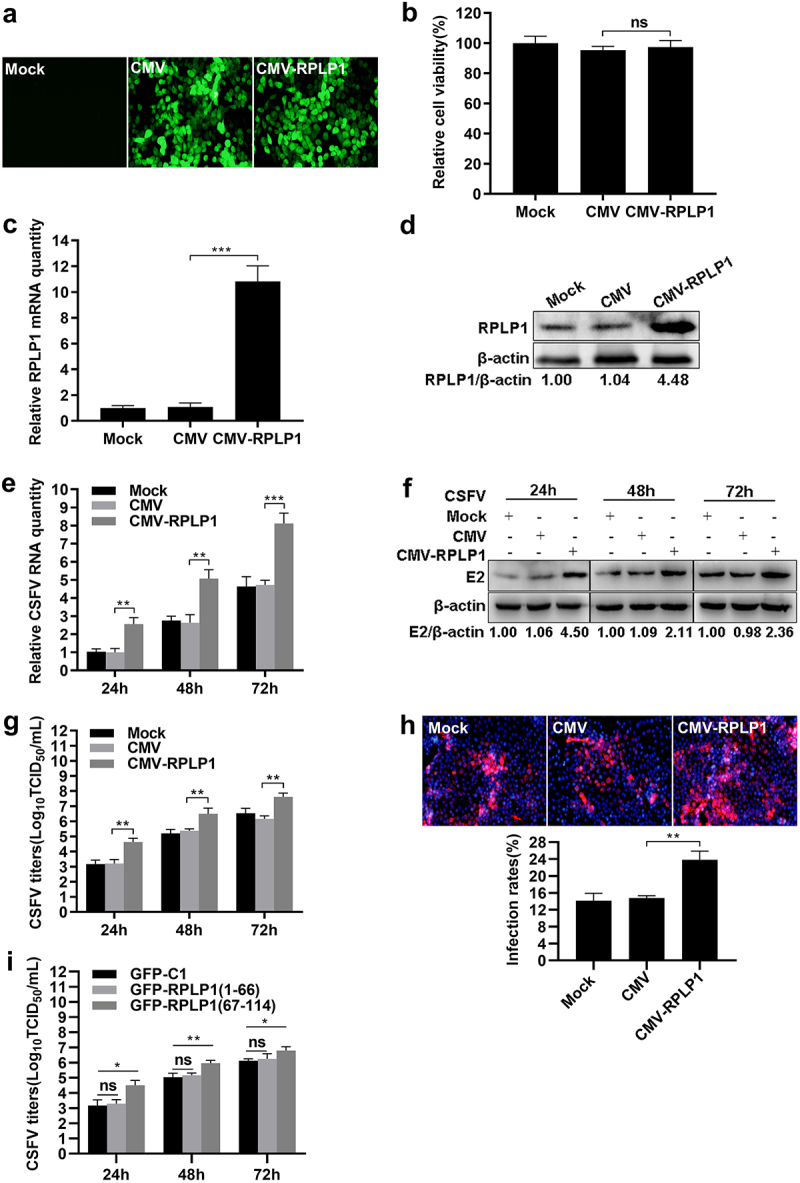
Overexpression of RPLP1 enhances CSFV proliferation.

To further verify the influence of RPLP1 on CSFV propagation, recombinant lentiviruses were used to construct RPLP1 overexpression (CMV-RPLP1) or control (CMV) cell lines, which were confirmed by fluorescence assays, mRNA detection, and WB analysis (Figure 4(a–d)). When the cells were infected with CSFV, in pace with increase of RPLP1, the expression levels of CSFV RNA and E2 were increased significantly at every checking time points in CMV-RPLP1 cells (Figure 4(e,f)). Moreover, overexpression of RPLP1 also significantly increased the progeny virus titers and viral infectivity on cells (Figure 4(g,h)). Considering the C-terminus of RPLP1 is responsible for the interaction with CSFV NS4B (Figure 1(e)), we subsequently detected whether overexpression the pivotal region had effect on CSFV production in PK15 cells. As expected, the aa 67 to 114 of RPLP1 contributed to progeny virus production at different time points, whereas the aa 1 to 66 did not (Figure 4(i)), suggesting the importance of the interaction for efficient virion production. Summarily, all these results further demonstrated RPLP1 plays an essential role in modulating CSFV proliferation.

(a) Confirmation the overexpression (CMV-RPLP1) and control (CMV) cell lines by detection of EGFP reporter. (b) Cell viability of RPLP1 overexpression cell lines was estimated by CCK-8. (c) The relative RPLP1 mRNA levels in RPLP1 overexpression cell lines were quantified by RT-qPCR. (d) The exogenous RPLP1 levels of RPLP1 overexpression cell lines were determined by WB and densitometry. The optical density ratio of RPLP1 to β-actin was calculated and normalized by the value of untransfected group (Mock). (e-g) Assessment of CSFV infection in RPLP1 overexpression cell lines. Cells were infected with CSFV (0.1 MOI) and collected at 24, 48, and 72 hpi for detection the viral RNA abundance, E2 protein expression, and progeny virus titers. (e) The relative CSFV RNA levels in RPLP1 overexpression cell lines were quantified by RT-qPCR. (f) The CSFV E2 protein expression levels in RPLP1 overexpression cell lines were determined by WB and densitometry. The optical density ratio of E2 protein to β-actin at each time points were measured and normalized by the values of untransfected group (Mock). (g) Progeny CSFV titers in RPLP1 overexpression cell lines were measured by IFA and expressed as TCID_50_/mL. (h) RPLP1 overexpression cell lines were infected with CSFV (0.1 MOI) and the representative immunofluorescence images were captured at 24 hpi. Cell nuclei (blue) were labeled with DAPI, and the CSFV E2 proteins (red) were stained with mouse anti-E2 mAb. The infection rate for CSFV was shown below the images, calculated by quantifying the number ratio of viral E2 positive cells in total DAPI-staining cells. At least 500 cells for each sample from three independent experiments were counted using ImageJ software. (i) The PK15 cells transiently transfected with pEGFP-RPLP1 (1–114), pEGFP-RPLP1 (1–66) or pEGFP-RPLP1 (67–114) for 36 h were infected with CSFV (0.1 MOI) and collected at 24, 48, and 72 hpi for detection the progeny virus titers. All results are shown as the mean ± SD of three independent experiments. Student’s t-test: ** (P < 0.01); *** (P < 0.001); ns (P > 0.05)

### CSFV proliferation modulates cellular RPLP1 expression

**Figure 5.**
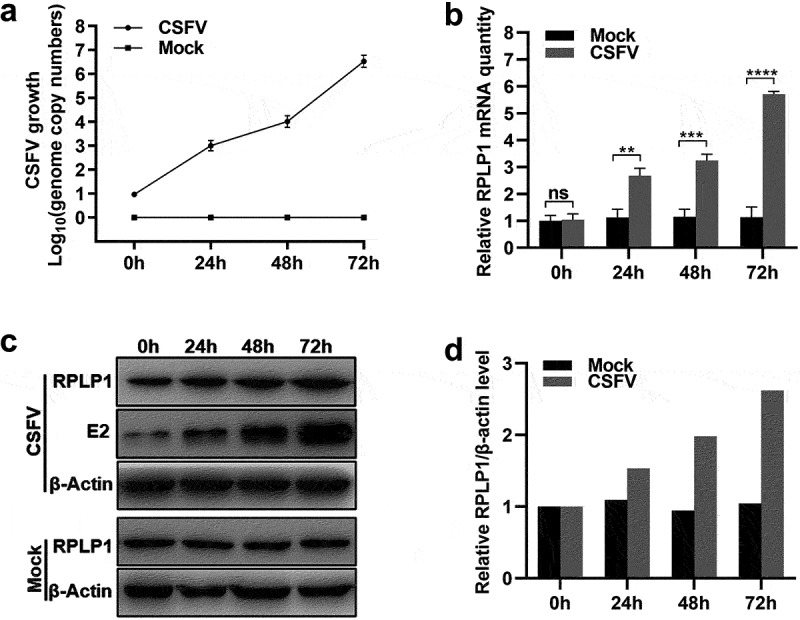
CSFV infection upregulates the expression of RPLP1.

As RPLP1 has an active effect on CSFV proliferation, we were wondering whether CSFV could modulate host RPLP1 expression to facilitate its replication. In Figure 5(a), the proliferative status of CSFV was quantified by its genome RNA copies after infection. The relative mRNA levels of RPLP1 in CSFV- or mock-infected cells were measured at different timepoints, and CSFV infection led to significant upregulation of RPLP1 mRNA within 24 hpi (Figure 5(b)). In consistent with the mRNA level, WB data clearly showed enhanced RPLP1 proteins in infected cells after CSFV infection which is confirmed by detection of viral E2 protein (Figure 5(c)). The semi-quantified RPLP1 protein level is shown in Figure 5(d). Together, these results clearly showed CSFV could up-regulate host RPLP1 expression to enhance virus growth.

PK15 cells were infected with 5 MOI of CSFV (or not infected) at 37°C for 1 h, followed by adding fresh medium. The cells were harvested at indicated time points (0, 24, 48, and 72 hpi). (a) The CSFV genome copies in PK15 cells were quantified at indicated time points by RT-qPCR. (b) The relative RPLP1 mRNA levels in PK15 cells were quantified by RT-qPCR and normalized to β-actin. (c) The CSFV E2 and RPLP1 protein levels in PK15 cells were detected by WB. (d) The RPLP1 protein levels in PK15 cells were analyzed by densitometry. The optical density ratio of RPLP1 to β-actin was calculated and the value of uninfected group (0 h) was normalized to 1.00. All results are shown as mean ± SD of three independent experiments. Student’s t-test: ** (P < 0.01); *** (P < 0.001); **** (P < 0.0001); ns (P > 0.05).

### RPLP1 has no regulatory effect on CSFV IRES efficiency

Now that RPLP1 interacts with CSFV NS4B and CSFV up-regulates host RPLP1 expression to enhance its propagation, how does RPLP1 enhance CSFV growth is worth exploring. Based on the function of RPLP1 in forming the ribosomal stalk and the dependency of CSFV genome translation on the IRES within 5′-UTR [5,33], we speculated that RPLP1 might involve in operation of CSFV IRES or CSFV RNA translation.

**Figure 6.**
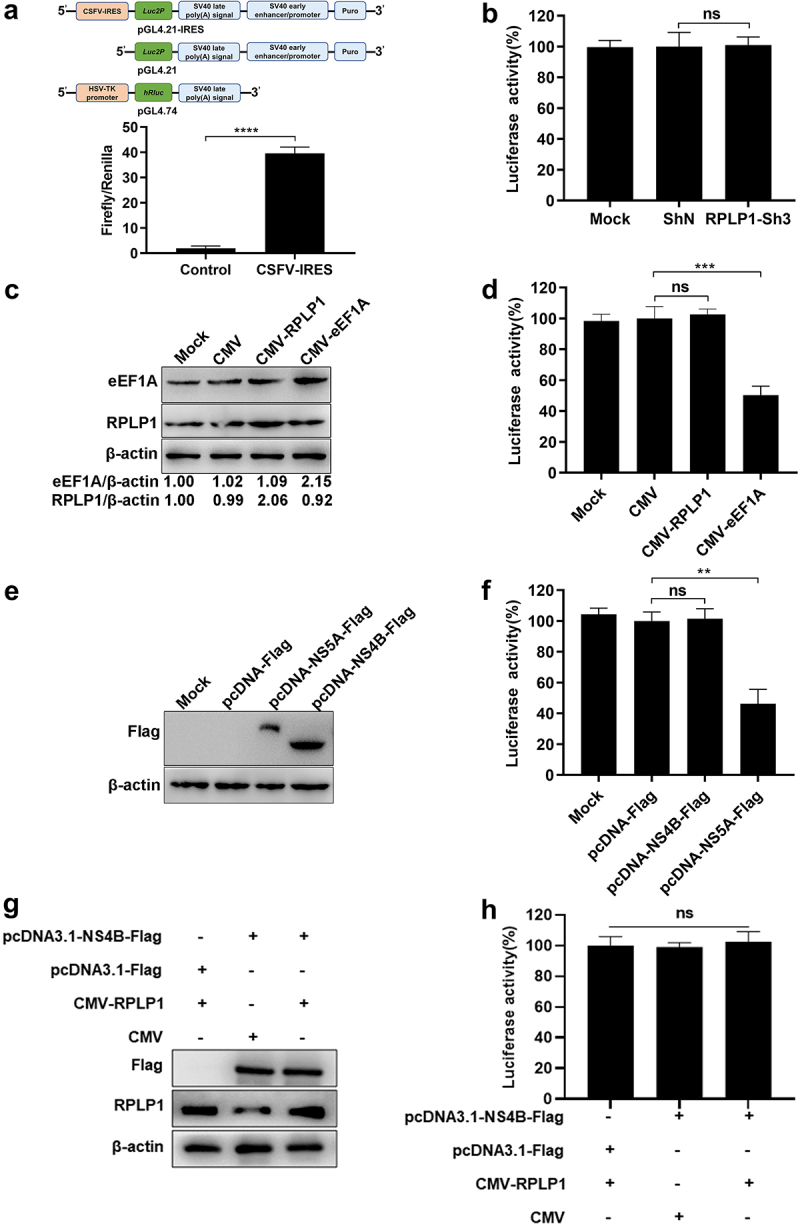
RPLP1 and NS4B take no significant effect on CSFV IRES activity.

Dual-luciferase reporter assay was applied to validate our hypothesis. The activity of CSFV IRES was confirmed by co-transfecting pGL4.21-IRES and pGL4.74 into PK15 cells, and results showed that the CSFV IRES operated successfully as expected (Figure 6(a)). Then, pGL4.21-IRES and pGL4.74 were co-transfected into the RPLP1 knockdown cell lines. The luciferase activity from cells with or without RPLP1 knockdown were comparable, which suggested depletion of RPLP1 did not affect CSFV IRES function (Figure 6(b)). Moreover, RPLP1 or NS4B was overexpressed to check their potential effects on CSFV IRES efficiency, with overexpressed eEF1A and NS5A, which were found to reduce the translation efficiency of CSFV IRES [34] serving as controls. In Figure 6(c–f), unlike in the controls which clearly showed repressed activity of CSFV IRES, overexpression of RPLP1 or NS4B had no significant effects on CSFV IRES efficiency. To further solidify our results, NS4B was overexpressed in the RPLP1 overexpression cell lines to detect the CSFV IRES efficiency. Figure 6(g,h) shows that simultaneous overexpression of PRLP1 and NS4B still made no difference in IRES-mediated translation efficiency. Taken together, these data indicated that RPLP1 and NS4B possess alternative mechanism rather than regulating CSFV IRES efficiency.

(a) Activity of CSFV IRES was detected by dual-luciferase reporter assay. In the upper part, constructs used for the assay were shown, including a CSFV IRES mediated firefly luciferase expression plasmid (pGL4.21-IRES) and a plasmid constitutively expressing renilla luciferase (pGL4.74), with a firefly luciferase plasmid lacking promoter used for control (pGL4.21). *Luc2P*: firefly luciferase, *hRluc*: synthetic renilla luciferase. In the lower part, the activity of CSFV IRES was quantified by measuring the activity ratio of firefly luciferase and co-transfected renilla luciferase (Firefly/Renilla). (b) Activity of CSFV IRES in cells with and without RPLP1 knockdown. The activity of CSFV IRES in cell lines stably expressing RPLP1-Sh3 (knockdown) or ShN (control) was measured as in a, and the IRES activity showed no significant difference with and without RPLP1 knockdown. (c) Verification the expression levels of RPLP1 and eEF1A in RPLP1 or eEF1A overexpression cell lines by WB and densitometry. (d) Activity of CSFV IRES in RPLP1 overexpression cells. Overexpression of eEF1A which suppresses IRES activity served as a control. (e) Verification the expression levels of NS4B and NS5A in PK15 cells at 36 hpt by WB. (f) Activity of CSFV IRES in NS4B and NS5A overexpression PK15 cells were detected, and the latter served as a positive control. (g) Verification the expression levels of NS4B and RPLP1 in RPLP1 overexpression cell lines at 36 hpt by WB. (h) Activity of CSFV IRES in NS4B and RPLP1 co-overexpression PK15 cells. All the results are shown as the mean ± SD of three independent experiments. Student’s t-test: ** (P < 0.01); *** (P < 0.001); **** (P < 0.0001); ns (P > 0.05).

### RPLP1 is essential for translation of CSFV genome

**Figure 7.**
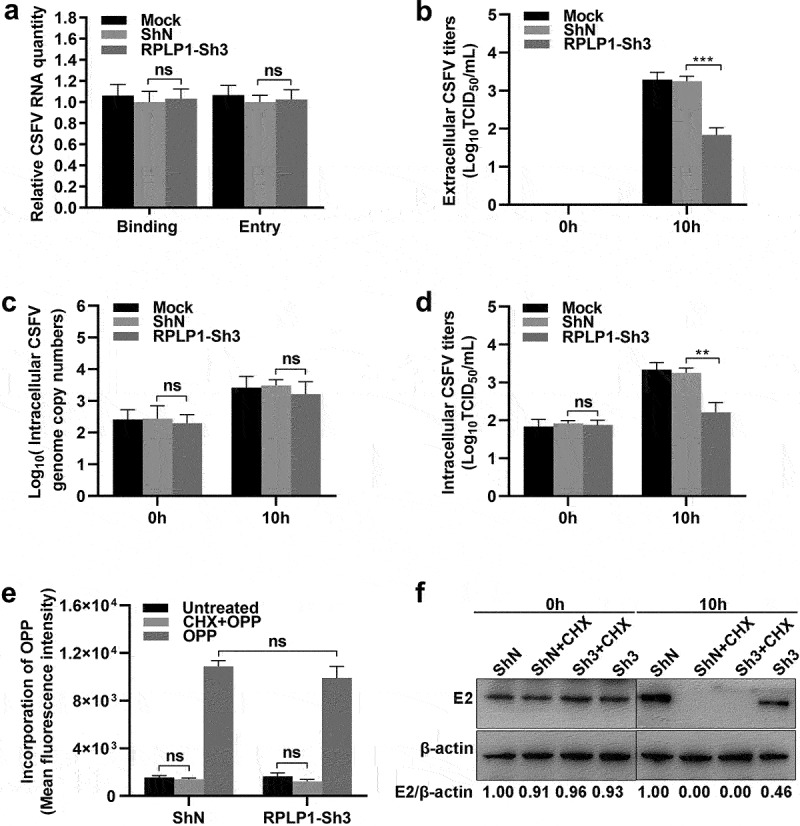
RPLP1 is essential for translation of CSFV RNA.

To further explore which stage of the CSFV life cycle RPLP1 participated in, virus binding and entry assays were firstly performed during CSFV infection. In Figure 7(a), the CSFV, which bound to cell surface or entered into cell, in RPLP1-Sh3 cells and ShN cells were comparable when quantified by the RNA genome, indicating that RPLP1 was not relating to the binding and entry stage of CSFV infection. Next, we analyzed intracellular viral genome copies and extracellular virus titers of CSFV in its first life cycle, which was measured to be 10 h previously [35]. Results showed that extracellular titers from RPLP1-Sh3 cells were decreased significantly compared with that from ShN cells (Figure 7(b)), but no statistically significant difference of the intracellular virus genome copies appeared in both groups (Figure 7(c)). We speculated that RPLP1 depletion hindered the release of progeny virus to supernatant or the production of intracellular infectious virus particles, which might lead to an accumulation or reduction of first generation of progeny virus within cells, respectively. To stress this issue, the intracellular virus titers within the first life cycle of CSFV were further detected. In Figure 7(d), intracellular virus titers were markedly reduced in RPLP1-Sh3 cells in comparison with that in ShN cells, indicating that RPLP1 functioned in production of CSFV particles. Considering the biological function of RPLP1 and its role in membrane protein translation [25], we speculated that RPLP1 might be important for the biogenesis of viral transmembrane structure proteins such as E2 protein of CSFV. Therefore, CSFV E2 envelope protein levels were measured under the condition of RPLP1 depletion. CHX, an inhibitor of global translation elongation [36], was used to block synthesis of new cellular and viral proteins. Besides, OPP, a chemical for nascent protein labeling in cell [37,38], was used to assess total protein synthesis with and without RPLP1 knockdown. As shown in Figure 7(e,f), depletion of RPLP1 had little to no inhibitory effect on global protein synthesis, while the synthesis of the E2 protein relied on RPLP1 strongly, depletion of which by RPLP1-Sh3 decreased the viral E2 protein level to less than half of the level in ShN control (Figure 7(f)). Our data revealed that RPLP1 is essential for translation of CSFV E2 protein, and this regulatory effect could at least partially explain the mechanism for impaired virion production and intracellular virus titers in RPLP1 depletion.

(a) The relative CSFV RNA levels was quantified at binding and entry steps. For binding assay, ShN or RPLP1-Sh3 cell lines were inoculated with 1 MOI of CSFV at 4°C for 1 h, unadsorbed virions were washed off and then the cells were harvested to detect the cell-bound viruses using RT-qPCR. For entry assay, after the binding step, cells were transferred to 37°C for 1 h to let the virus internalize, then washed and harvested for viral RNA quantification. (b) Extracellular viral titers were measured in the first round of life cycle (10 hpi). In comparison to the control (ShN and mock), RPLP1 knockdown (RPLP1-Sh3) reduced the extracellular viral titer significantly. (c) Intracellular CSFV genome copies were measured in the first round of virus life cycle (10 hpi), and no difference was observed with (RPLP1-Sh3) and without (ShN and mock) knockdown of RPLP1. (d) Intracellular viral titers were measured at 10 hpi. Knockdown of RPLP1 (RPLP1-Sh3) significantly reduced infectious viral titers, suggesting that RPLP1 facilitate production of infectious virions in the cell. (e) Total cellular protein synthesis was quantified by incorporation of OPP. While incorporation of OPP was blocked by cycloheximide (CHX) as it suppressed translation of all mRNAs (CHX+OPP), RPLP1 knockdown did not affect global protein translation as there was no difference between the RPLP1 knockdown (RPLP1-Sh3) and control (ShN). (f) The CSFV E2 protein expression levels were evaluated by WB and densitometry. RPLP1-Sh3 and ShN cell lines treated with 100 μg/mL CHX starting 0.5 h before CSFV infection were used as translation inhibition control. The optical density ratio of E2 protein to β-actin were calculated and the values of control group (ShN) were normalized to 1.00. All the results are shown as the mean ± SD of three independent experiments. Student’s t-test: ** (P < 0.01); *** (P < 0.001); ns (P > 0.05).

## Discussion

The interaction between host RPs and diverse viruses to regulate viral infection has been widely studied in recent years. Generally, the functional characteristics of interactivity of host RPs with viral proteins can be sketchily classified into three categories as follows: (i) working as viral receptor; (ii) being relation to viral replication and assembly; and (iii) promoting viral translation [13]. For example, RPS2 works as a membrane receptor binding to viral envelope protein E in the infection of DENV and YFV [39]. RPL22 is hijacked by Herpes simplex virus type 1 (HSV-1) via interacting with its ICP4 protein to regulate viral DNA replication [40]. RPL7 is a binding partner of human immunodeficiency virus type 1 (HIV-1) Gag, which possesses a powerful DNA/RNA chaperone activity contributing to virions assembly [41]. RPS25 acts an essential part in viral IRES-mediated translation, once it was depleted, the propagation of Hepatitis C virus (HCV) and poliovirus were impaired [42]. The RPS19 interacts with N protein of Sin nombre hantavirus (SNV) to configurate the 43S pre-initiation complex and directly mediate translation initiation of viral RNA [43,44]. RPL18, is one of the well-known RPs, which interacts with many viral proteins to mediate viral translation in different mechanisms. The interaction of RPL18 with Cauliflower mosaic virus (CaMV) P6 in complex consisting of eIF3 and several other RPs are required for viral translational transactivation [45,46]. Besides, RPL18 interacts with N protein of Rice stripe tenuivirus (RSV) and NS1 protein of DENV, depletion of RPL18 effectively inhibits replication and translation of these viruses [47,48]. RPLP1, which is not absolutely required for global cellular translation or cell viability but may regulate the translation of a specific subset of transcripts, was identified as one of CSFV NS4B binding partners in our previous study [11,49,50]. Herein, our results of co-IP, GST-pulldown, and confocal microscopy assays also exhibited that RPLP1 associated with NS4B to form complexes (Figures 1 and 2), which further demonstrated the interaction between host RPLP1 and NS4B.

Regardless of their positive or negative roles, host factors interacting with proteins of CSFV are usually involved in virus proliferation [51]. Rab18 is necessary for replication and assembly steps of CSFV via interacting with viral NS5A [52]. RPS20 interacts with CSFV N^pro^ and inhibited virus replication by modulating TLR3 expression [53]. Ferritin heavy chain (FHC) interacts with CSFV NS4B, enhances CSFV replication, and works positively in antagonizing apoptosis by regulating the production of cellular ROS [8]. Fatty acid synthase (FASN) interacts with CSFV NS4B and facilitates the virus proliferation by regulating the formation of lipid droplets (LDs) [54]. Recently, tumor susceptibility gene 101 (TSG101) has been reported to promote CSFV replication by interacting with NS4B and NS5B to form replication complexes [55]. In current study, CSFV production was apparently improved by overexpression of RPLP1 (Figure 4); in contrast, it was drastically diminished by recombinant lentivirus-mediated RPLP1 knockdown (Figure 3), which indicated that RPLP1 could positively modulate CSFV propagation. Although the exact mechanism of this modulation remains elusive, our data showing the C-terminal region of RPLP1 interacts with CSFV NS4B, and this part is also sufficient to promote virus production, supports the idea that the interaction between RPLP1 and NS4B may involves in the regulation of CSFV proliferation.

Owing to its limited coding capabilities of genome, CSFV usually regulates the expression of host proteins to affect viral replication. Heat shock protein 70 (HSP70) and caveolin-1, which have been reported to enhance CSFV propagation, are revealed to be up-regulated during infection [56,57]. Accordingly, tumor necrosis factor receptor-associated factor 6 (TRAF6) and RPS20, which have been reported to inhibit CSFV replication, are down-regulated by CSFV [11,58]. Our results also showed that infection of CSFV increased cellular RPLP1 expression (Figure 5), which could be benefit to enhance CSFV propagation due to the positive role of RPLP1 in CSFV growth. Similar to our findings, RPLP1 was also observed to be significantly up-regulated after HSV-1 infection in L-02 cells [59]. However, whether the up-regulation of RPLP1 expression in CSFV-infected cells depends on NS4B needs more study.

With regard to the underlying mechanism of RPLP1 enhancing the proliferation of CSFV, we first showed that RPLP1 was not in relation to translation efficiency of CSFV IRES (Figure 6). This is identical to an earlier study which demonstrated the activity of HCV IRES was not affected by RPLP1 depletion [60]. Next, we analyzed the amount of CSFV binding to cell surface or entered into cell (Figure 7(a)), and results indicated that RPLP1 was not involved in absorb and entry stage of CSFV. Moreover, a further analysis of CSFV infection within the first life cycle showed that RPLP1 depletion led to significantly reduce both the extracellular and intracellular virus titers, as well as E2 protein expression, but had no effects on intracellular virus genome replication and cellular global protein synthesis (Figure 7(b–f)). Combined with the above results, we concluded that RPLP1 is involved in translating of CSFV RNA rather than viral RNA replication. This is in accordance with three recent reports which demonstrated the requirement of RPLP1 in protein biogenesis of DENV,YFV and ZIKV, another three members of family *Flaviviridae* [24,25,61].

In conclusion, we demonstrated that RPLP1 interacts with CSFV NS4B and positively enhances CSFV production via promoting translation of viral genome. This study would help to deepen our understanding of the molecular basis of CSFV infection in host cells and provides a potential therapeutic target for prevention and control of CSF.

## Data Availability

The data that support the findings of this study are available from the corresponding author, W. D., upon reasonable request.
